# Quantitative drug susceptibility testing for Mycobacterium tuberculosis using unassembled sequencing data and machine learning

**DOI:** 10.1371/journal.pcbi.1012260

**Published:** 2024-08-05

**Authors:** The CRyPTIC consortium

**Affiliations:** University of Oxford, Oxford, United Kingdom; University of Rome La Sapienza: Universita degli Studi di Roma La Sapienza, ITALY

## Abstract

There remains a clinical need for better approaches to rapid drug susceptibility testing in view of the increasing burden of multidrug resistant tuberculosis. Binary susceptibility phenotypes only capture changes in minimum inhibitory concentration when these cross the critical concentration, even though other changes may be clinically relevant. We developed a machine learning system to predict minimum inhibitory concentration from unassembled whole-genome sequencing data for 13 anti-tuberculosis drugs. We trained, validated and tested the system on 10,859 isolates from the CRyPTIC dataset. Essential agreement rates (predicted MIC within one doubling dilution of observed MIC) were above 92% for first-line drugs, 91% for fluoroquinolones and aminoglycosides, and 90% for new and repurposed drugs, albeit with a significant drop in performance for the very few phenotypically resistant isolates in the latter group. To further validate the model in the absence of external MIC datasets, we predicted MIC and converted values to binary for an external set of 15,239 isolates with binary phenotypes, and compare their performance against a previously validated mutation catalogue, the expected performance of existing molecular assays, and World Health Organization Target Product Profiles. The sensitivity of the model on the external dataset was greater than 90% for all drugs except ethionamide, clofazimine and linezolid. Specificity was greater than 95% for all drugs except ethambutol, ethionamide, bedaquiline, delamanid and clofazimine. The proposed system can provide quantitative susceptibility phenotyping to help guide antimicrobial therapy, although further data collection and validation are required before machine learning can be used clinically for all drugs.

## Author summary

Tuberculosis is a leading cause of morbidity and mortality globally, killing over 1.5 million people each year. Tuberculosis drug susceptibility testing is used to determine which antibiotics should be used to manage the disease, particularly in view of the rise in multidrug resistant tuberculosis. The current standard for testing, binary phenotypes, only capture changes in minimum inhibitory concentration when these cross a critical concentration, even though other changes may be clinically relevant. We developed a machine learning system to predict minimum inhibitory concentration directly from sequencing data for 13 antibiotics. We find that the model has an accuracy of 92% for first-line drugs, 91% for fluoroquinolones and aminoglycosides, and 90% for new and repurposed drugs, with a significant drop in performance for resistant isolates in the latter group. We validated the model on an external dataset with binary phenotypes. The sensitivity of the model on the external dataset was greater than 90% for all drugs except ethionamide, clofazimine and linezolid. Specificity was greater than 95% for all drugs except ethambutol, ethionamide, bedaquiline, delamanid and clofazimine. The proposed system could help guide antimicrobial therapy. However, further data and validation are required before machine learning can be used clinically for all drugs.

## Background

In 2020, 10 million individuals fell ill with tuberculosis and 1.5 million died from the infection [1]. The problem of multidrug-resistant tuberculosis (MDR-TB)–defined as resistance to isoniazid and rifampicin–has been described by the World Health Organization (WHO) as a global health crisis [1]. Despite advances in diagnostics and treatment, MDR-TB remains under-detected and treatment success remains stubbornly below 60% globally [1]. The SARS-CoV-2 pandemic has likely set back the progress that has been made by years [2].

The WHO has called for universal tuberculosis drug susceptibility testing [3], but two major barriers remain for universal susceptibility testing in the face of rapidly evolving resistance. First, culture-based susceptibility testing is too slow, infra-structure dependent (biosafety level 3 laboratories are required), and technically challenging to offer a realistic solution [4]. Novel molecular assays have been developed to rapidly detect resistance to a subset of first- and second-line drugs, but are limited in the number of resistance-conferring mutations they can detect [5], constraining their sensitivity. Some countries now rely on whole-genome sequencing (WGS) to identify susceptibility to first-line drugs [6], but suffer the same limitations in the number of resistance-conferring mutations detected, as well as financial and technical implementation requirements. Second, culture-based susceptibility testing typically generates binary phenotypes, where susceptible isolates are differentiated from resistant ones based on critical concentrations of antibiotic that are not always firmly relatable to clinical outcome. Semi-quantitative measures of drug susceptibility such as minimum inhibitory concentrations (“MICs”) could be more clinically useful as they lend themselves to the future development of personalized dosing regimens, by indicating whether it might be possible to overcome ‘resistance’ through higher drug dosing.

Machine learning algorithms have accurately predicted binary susceptibility from whole genome sequencing data in *Mycobacterium tuberculosis* [7–9], and predicted MICs in other pathogens like *Salmonella* [10], *N*. *gonorrhea* [11], *S*. *aureus* [12], and *E*. *coli* [13], using both genomic mutations [7–9,11,13] and *k*-mers [10,12] as data inputs. The difficulty in generating semi-quantitative phenotypic data for *M*. *tuberculosis* has meant that machine learning approaches have yet to be applied to the problem of predicting such phenotypes from WGS data for this major pathogen.

Here, we assess how well a machine learning system can use genome-wide genomic features to predict MICs for 13 anti-tuberculosis drugs, including for bedaquiline and delamanid for which good molecular assays are currently lacking. We leverage a new, recently published dataset from CRyPTIC (a consortium working on genotypic / phenotypic associations for drug resistance in *M*. *tuberculosis*) [14], and adapt previously described extreme gradient boosting machine models for use on genome-wide *k*-mers directly from unassembled sequencing reads. We then assess whether MIC predictions can be used to accurately predict the more commonly generated binary phenotypes from Mycobacterial Growth Indicator Tubes (MGIT) in an independent dataset.

## Results

### Dataset characteristics

The CRyPTIC dataset (Table 1) included MICs for 10,859 isolates from 22 countries. Lineages 1 to 4, 6 and *Mycobacterium bovis* were represented, with lineage 4 (50%, 5,436/10,859) and lineage 2 (35%, 3,745/10,859) the most common (Table A in S1 Appendix). About 28% of samples (3,033/10,859) were multi-drug resistant. MICs were determined using a 96-well broth microdilution plate, and were available for three first-line antibiotics (isoniazid, rifampicin, ethambutol), plus rifabutin, two fluoroquinolones (moxifloxacin, levofloxacin), two injectable agents (amikacin, kanamycin), ethionamide, two newer drugs (bedaquiline, delamanid) and two repurposed drug (linezolid, clofazimine). Pyrazinamide was not present on the microdilution plate for technical reasons. We present results for kanamycin and rifabutin exclusively in Table B in S1 Appendix as they are less commonly prescribed.

**Table 1:** Sample provenance and resistance profiles in the CRyPTIC dataset.

	Isolate origin	Isolates resistance to each drug													
	Country	Total	INH	RIF	EMB	MXF	LEV	AMI	KAN	ETH	RIF	BDQ	CFZ	DLM	LZD
1	Algeria	24	2	2	0	0	0	0	0	2	1	0	0	0	0
2	Belarus	101	54	41	14	6	9	10	14	17	43	0	1	1	0
3	Brazil	331	98	72	22	7	6	10	3	37	70	2	1	3	3
4	China	901	256	217	138	92	105	34	38	60	202	9	18	17	9
5	Germany	691	206	146	27	25	39	20	42	41	149	1	0	3	6
6	India	1259	625	612	388	302	317	77	94	198	561	5	1	15	14
7	Italy	843	352	318	63	62	89	47	81	102	304	10	8	11	4
8	Japan	1	1	1	0	0	0	0	0	1	1	0	0	0	0
9	Kyrgyzstan	26	23	13	3	3	8	6	8	7	16	0	0	0	0
10	Nepal	197	138	121	48	76	111	3	6	42	132	0	0	0	0
11	Pakistan	365	295	276	81	45	106	16	15	39	245	6	7	3	3
12	Peru	2.605	1083	782	326	126	135	144	175	264	597	3	0	4	0
13	Slovenia	1	0	0	0	0	0	0	0	0	0	0	0	0	0
14	Somalia	2	0	0	0	0	0	0	0	0	0	0	0	0	0
15	South Africa	1,978	698	538	257	206	200	194	244	260	531	31	58	12	25
16	South Georgia	1	1	0	0	0	0	0	0	0	0	0	0	0	0
17	Sweden	5	5	5	3	3	4	1	1	4	5	0	0	0	0
18	Tajikistan	17	13	9	2	2	3	2	3	0	10	0	0	0	0
19	Turkmenistan	109	79	56	2	8	11	8	11	5	67	0	0	0	0
20	Ukraine	28	15	15	0	4	5	3	7	3	14	0	0	0	0
21	United States	125	99	80	25	10	9	1	3	19	96	0	0	1	2
22	Vietnam	1249	453	186	61	11	17	10	4	86	202	2	11	11	7
	**Total**	**10,859**	**4,496**	**3,490**	**1,460**	**988**	**1,174**	**586**	**749**	**1,187**	**3,246**	**69**	**105**	**81**	**73**

INH: Isoniazid, RIF: Rifampin, EMB: Ethambutol, MXF: Moxifloxacin, LEV: Levofloxacin, AMI: Amikacin, KAN: Kanamycin, ETH: Ethionamid, RFB: Rifabutin, BDQ: Bedaquilin, DLM: Delamanid, CFZ: Clofazimine, LZD: Linezolid.

### Minimum inhibitory concentration prediction

**Fig 1:**
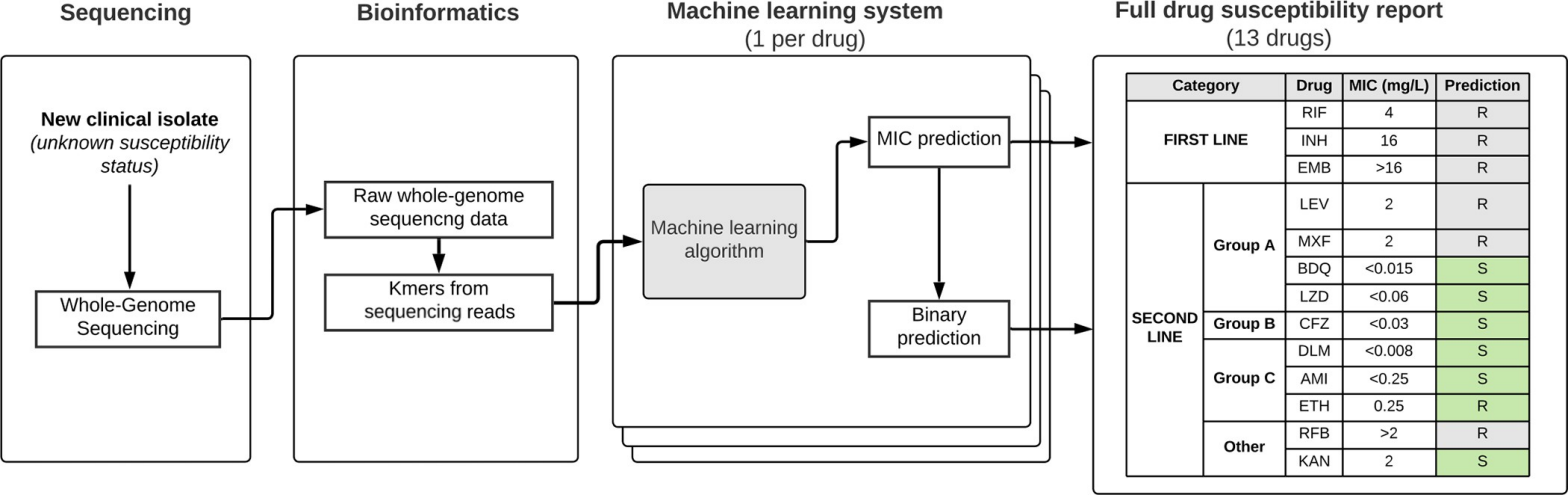
Illustration of the machine learning system workflow to predict MIC and drug susceptibility status for 13 antibiotics using sequencing data. Drug names: INH: Isoniazid, RIF: Rifampin, EMB: Ethambutol, MXF: Moxifloxacin, LEV: Levofloxacin, AMI: Amikacin, KAN: Kanamycin, ETH: Ethionamid, RFB: Rifabutin, BDQ: Bedaquilin, CFZ: Clofazimine, DLM: Delamanid, LZD: Linezolid. Groups: Guidelines and drug groupings refer to World Health Organization Consolidated Guidelines on Tuberculosis.

We developed a *k-*mer-based, hypothesis-free, genome-wide supervised machine learning algorithm to predict MICs for 13 antibiotics (as described in Methods and presented in Fig 1). To assess the performance of the machine learning system on the widest possible set of antibiotics, it was initially trained on 75% of the CRyPTIC dataset (8,146 randomly selected isolates). Predictions were made for the remaining 25% (2,713 isolates). We generated confidence matrices for each drug (S1 Fig) and calculated essential agreement rates (when the MIC is correctly predicted within one doubling dilution) (Table 2).

**Table 2:** Essential agreement rates (within 1 doubling dilution) for all drugs.

	All isolates			Phenotypically susceptible			Phenotypically resistant			Comparison (R vs S)	
Drug	n	EA	95% CI	n	EA	95% CI	n	EA	95% CI	Difference	P-value
INH	2,380	0.942	(0.932–0.951)	1,207	0.978	(0.969–0.986)	1,173	0.905	(0.888–0.921)	0.073	<0.001
EMB	1,907	0.950	(0.940–0.960)	1,501	0.952	(0.941–0.963)	406	0.943	(0.921–0.966)	0.009	0.48
RIF	2,246	0.929	(0.919–0.940)	1,315	0.926	(0.912–0.940)	931	0.933	(0.917–0.949)	-0.007	0.51
LEV	1,936	0.949	(0.940–0.959)	1,626	0.970	(0.962–0.979)	310	0.839	(0.798–0.880)	0.131	<0.001
MXF	1,699	0.909	(0.895–0.922)	1,438	0.920	(0.906–0.934)	261	0.847	(0.803–0.890)	0.073	<0.001
AMI	2,248	0.975	(0.968–0.981)	2,090	0.982	(0.977–0.988)	158	0.873	(0.822–0.925)	0.109	<0.001
KAN	2,297	0.982	(0.976–0.987)	2,109	0.989	(0.985–0.994)	188	0.899	(0.856–0.942)	0.090	<0.001
ETH	2,210	0.913	(0.901–0.924)	1,901	0.941	(0.930–0.951)	309	0.741	(0.692–0.790)	0.200	<0.001
RFB	2,505	0.935	(0.925–0.944)	1,659	0.969	(0.961–0.978)	846	0.866	(0.844–0.889)	0.103	<0.001
BDQ	2,130	0.933	(0.923–0.944)	2,114	0.940	(0.930–0.950)	16	0.062	(0.000–0.181)	0.878	<0.001
CFZ	1,917	0.961	(0.953–0.970)	1,894	0.971	(0.964–0.979)	23	0.130	(0.000–0.268)	0.841	<0.001
LZD	1,805	0.936	(0.924–0.947)	1,777	0.944	(0.934–0.955)	28	0.393	(0.212–0.574)	0.551	<0.001
DLM	1,990	0.964	(0.956–0.972)	1,970	0.973	(0.966–0.980)	20	0.100	(0.000–0.231)	0.873	<0.001
**Mean (weighted)**		0.945			0.959			0.874		0.025	
**Std (weighted)**		0.021			0.021			0.108			

Drug names: INH: Isoniazid, RIF: Rifampin, EMB: Ethambutol, MXF: Moxifloxacin, LEV: Levofloxacin, AMI: Amikacin, KAN: Kanamycin, ETH: Ethionamid, RFB: Rifabutin. BDQ: Bedaquilin. DLM: Delamanid. CFZ: Clofazimine. LZD: Linezolid. Other acronyms: EA: Essential Agreement. CI: Confidence interval. R: resistance. S: Susceptible. std: Standard deviation. Notes: p-values calculated using McNemar’s chi square.

For the first-line drugs, the essential agreement rate was 94.2% for isoniazid (2,242/2,380), 95.0% for ethambutol (1,811/1,907), and 92.9% for rifampicin (2,086/2,246) (Table 2). We compared essential agreement rates between phenotypically resistant and susceptible isolates (after converting MICs to binary phenotypes using previously defined epidemiological cutoffs [15]) to see if the predictions made by the machine learning model were more accurate for one of the two subgroups. There was no statistically significant difference in essential agreement between the two subgroups for ethambutol and rifampicin. There was a 7.3% decrease in essential agreement for phenotypically resistant samples compared to susceptible isolates for isoniazid (97.8% *vs* 90.5%, p<0.001). Perfect agreement rates (exact MIC predicted correctly) were on average 30.1% lower than essential agreement rates across drugs (p<0.001) and are presented in Table B in S1 Appendix.

Essential agreement was 94.9% for levofloxacin (1,837/1,936), 90.9% for moxifloxacin (1,544/1,699), 97.5% for amikacin (2,191/2,248), and 91.3% for ethionamide (2,017/2,210). Essential agreement was lower for phenotypically resistant than for phenotypically susceptible isolates for all drugs, with differences of 13.1%, 7.3%, 10.9%, and 20.0%, for levofloxacin, moxifloxacin, amikacin, and ethionamide, respectively (p<0.001). We note that there were fewer phenotypically resistant isolates for these four drugs: 310, 261, 158, and 309, respectively, in the CRyPTIC dataset–which could explain the challenge in lower accuracy. EA was above 93% for all new and repurposed drugs: 93.3% for bedaquiline (1,987/2,130), 96.1% for clofazimine (1,842/1,917), 93.6% for linezolid (1,689/1,805), and 96.4% (1,918/1,990) for delamanid. Essential agreement dropped significantly for phenotypically resistant isolates: 39.3% (11/28) for linezolid, 13.0% for clofazimine (3/23), 10.0% for delamanid (2/20), and 6.2% for bedaquiline (1/16), demonstrating the poor predictive performance for these drugs, with the high essential agreement due to the majority of susceptible predictions. We observe the bimodal distribution of MICs for rifampicin, rifabutin, amikacin, and isoniazid, while those for ethambutol, kanamycin, moxifloxacin, and levofloxacin show a more unimodal distribution, correlating with less accurate predictions distributed across a broader range of the confusion matrix (S1 Fig) and consistent with previous findings in the CRyPTIC dataset [15]. Results by lineage are presented in Table C in S1 Appendix.

### Binarising MICs

We assessed whether the machine learning model could be used to predict binary susceptibility status, given binary predictions remain the standard by which most patients are treated. We begin by considering the MIC value predicted by the model as a probability of resistance, where an increase in predicted MIC corresponds to increase in probability of resistance. For example, if all MIC predictions range from 1 to 8, then a prediction of 8 is considered to be a 100% likelihood of resistance and prediction of 1 to a 0% likelihood of resistance, for the purpose of generating a receiver operating characteristic (ROC) curve and of calculating an area under the curve (AUC) metric (Table 3). For first-line drugs, AUC was 0.983 for isoniazid (95% CI 0.979–0.987), 0.992 for rifampicin (0.988–0.994), and 0.965 for ethambutol (0.957–0.973). For second-line drugs, AUC was 0.945 for levofloxacin (0.931–0.963), 0.970 for moxifloxacin (0.958–0.982), 0.970 for amikacin (0.953–0.984), 0.960 for kanamycin (0.941–0.975), and 0.939 for ethionamide (0.924–0.952). For new and repurposed drugs, AUCs were lower—0.908 for bedaquiline (0.876–0.952), 0.879 for clofazimine (0.832–0.934), 0.781 for linezolid (0.695–0.866) and 0.785 for delamanid (0.711–0.861). However, we note that these four results are likely significant overestimations due to the very low number of true phenotypic positives in the test set (between 14 and 19).

**Table 3:** Binarised performance of the machine learning system on the CRyPTIC dataset and on an independent dataset.

Drug	Dataset	AUC	AUC_CI	Sens	Sens CI	Spec	Spec_CI	PPV	PPV_CI	NPV	NPV_CI	Acc
Drugs present in both the CRyPTIC dataset and the external validation dataset												
INH	CRyPTIC	0.983	(0.979,0.987)	0.950	(0.937–0.963)	0.981	(0.969–0.993)	0.980	(0.972–0.988)	0.953	(0.942–0.964)	0.966
INH	Seq&Treat	0.971	(0.967,0.975)	0.936	(0.927–0.945)	0.963	(0.958–0.968)	0.899	(0.89–0.908)	0.977	(0.975–0.979)	0.956
RIF	CRyPTIC	0.992	(0.988,0.994)	0.971	(0.96–0.982)	0.973	(0.964–0.982)	0.962	(0.95–0.974)	0.979	(0.973–0.985)	0.972
RIF	Seq&Treat	0.981	(0.978,0.984)	0.960	(0.953–0.967)	0.984	(0.98–0.988)	0.944	(0.936–0.952)	0.988	(0.987–0.989)	0.978
LEV	CRyPTIC	0.945	(0.931,0.963)	0.916	(0.884–0.948)	0.961	(0.947–0.975)	0.816	(0.779–0.853)	0.984	(0.981–0.987)	0.954
LEV	Seq&Treat	0.949	(0.931,0.968)	0.912	(0.858–0.966)	0.925	(0.89–0.96)	0.839	(0.779–0.899)	0.961	(0.946–0.976)	0.921
MXF	CRyPTIC	0.970	(0.958,0.982)	0.950	(0.923–0.977)	0.961	(0.95–0.972)	0.816	(0.777–0.855)	0.991	(0.989–0.993)	0.959
MXF	Seq&Treat	0.948	(0.930,0.963)	0.916	(0.884–0.948)	0.951	(0.939–0.963)	0.729	(0.691–0.767)	0.988	(0.986–0.99)	0.947
AMI	CRyPTIC	0.970	(0.953,0.984)	0.918	(0.873–0.963)	0.991	(0.979–1)	0.884	(0.838–0.93)	0.994	(0.993–0.995)	0.986
AMI	Seq&Treat	0.924	(0.899,0.950)	0.877	(0.838–0.916)	0.949	(0.936–0.962)	0.673	(0.635–0.711)	0.985	(0.983–0.987)	0.941
KAN	CRyPTIC	0.960	(0.941,0.975)	0.910	(0.867–0.953)	0.970	(0.958–0.982)	0.728	(0.679–0.777)	0.992	(0.991–0.993)	0.965
KAN	Seq&Treat	0.882	(0.858,0.914)	0.820	(0.757–0.883)	0.875	(0.855–0.895)	0.413	(0.379–0.447)	0.978	(0.976–0.98)	0.870
ETH	CRyPTIC	0.939	(0.924,0.952)	0.874	(0.834–0.914)	0.894	(0.878–0.91)	0.572	(0.538–0.606)	0.978	(0.975–0.981)	0.891
ETH	Seq&Treat	0.812	(0.726,0.894)	0.906	(0.8–1)	0.667	(0.569–0.765)	0.630	(0.516–0.744)	0.919	(0.839–0.999)	0.759
Drugs only present in the CRyPTIC dataset												
EMB	CRyPTIC	0.965	(0.957,0.973)	0.931	(0.905–0.957)	0.894	(0.88–0.908)	0.704	(0.671–0.737)	0.980	(0.976–0.984)	0.902
RFB	CRyPTIC	0.988	(0.985,0.991)	0.955	(0.941–0.969)	0.957	(0.947–0.967)	0.918	(0.901–0.935)	0.977	(0.972–0.982)	0.956
BDQ	CRyPTIC	0.908	(0.876,0.952)	0.875	(0.702–1)	0.760	(0.744–0.776)	0.027	(0.025–0.029)	0.999	(0.999–0.999)	0.761
CFZ	CRyPTIC	0.879	(0.832,0.934)	0.826	(0.656–0.996)	0.722	(0.702–0.742)	0.035	(0.032–0.038)	0.997	(0.997–0.997)	0.724
LZD	CRyPTIC	0.781	(0.695,0.866)	0.571	(0.329–0.813)	0.876	(0.851–0.901)	0.068	(0.059–0.077)	0.992	(0.992–0.992)	0.871
DLM	CRyPTIC	0.785	(0.711,0.861)	0.750	(0.531–0.969)	0.724	(0.702–0.746)	0.027	(0.025–0.029)	0.997	(0.997–0.997)	0.724

Drug names: INH: Isoniazid, RIF: Rifampin, EMB: Ethambutol, MXF: Moxifloxacin, LEV: Levofloxacin, AMI: Amikacin, KAN: Kanamycin, ETH: Ethionamid, RFB: Rifabutin. BDQ: Bedaquilin. DLM: Delamanid. CFZ: Clofazimine. LZD: Linezolid. Other acronyms: EA: Essential Agreement. CI: Confidence interval. R: resistance. S: Susceptible. AUC: Area under the Curve. Sens: Sensitivity. Spec: Specificity. PPV: Positive Predictive Value. NPV: Negative Predictive Value. Acc: Accuracy

We then converted the MIC predictions to binary predictions and compared them to the binary phenotypes determined using previously defined epidemiological cutoffs [15] (Methods, Table D in S1 Appendix). For first-line drugs, sensitivity of the machine learning model was 95.0% for isoniazid, 97.1% for rifampicin, and 93.1% for ethambutol, with a specificity of 98.1%, 89.4%, and 97.3%, respectively. Sensitivity was 91.6% for levofloxacin, 95.0% for moxifloxacin, 91.8% for amikacin, and 87.4% for ethionamide, with a specificity of 96.1%, 96.1%, 99.1%, and 89.4%, respectively. For new and repurposed drugs, we find a sensitivity of 87.5% for bedaquiline, 82.6% for clofazimine, 57.1% for linezolid, and 75.0% for delamanid, with a specificity of 76.0%, 72.2%, 87.6%, and 72.4%, respectively (Table 3).

### External validation and comparison to diagnostic standards

No dataset yet exists to validate the performance of MIC predictions for the full spectrum of drugs on the CRyPTIC plate. In order to measure the performance of our predictor on an external dataset, we assess how a model trained on the entire CRyPTIC dataset can predict binary resistance in a separate set of 15,577 isolates for first-line and second-line drugs from the Seq&Treat consortium whose data, along with CRyPTIC’s, were used to generate the WHO catalogue of mutations associated with drug resistance in *M*. *tuberculosis* [16] (Table A in S1 Appendix). AUC was 0.971 for isoniazid (95%CI 0.967–0.975) and 0.981 for rifampicin (0.978–0.984), in both cases 1.1% lower than in CRyPTIC (p<0.01). Sensitivity was 93.6% for isoniazid and 96.0% for rifampicin, with a specificity of 99.2% and 99.0%, respectively. For second-line drugs, AUC was higher than CRyPTIC for levofloxacin (0.949), and lower for moxifloxacin (0.948), amikacin (0.924), and ethionamide (0.812). Sensitivity was 91.2%, 91.6%, 87.7%, and 90.6%, respectively, with a specificity of 92.5%, 95.1%, 94.9%, and 66.7%, respectively (Tables C and E in S1 Appendix).

We next used the independent set to compare predictions from the machine learning system with those from a validated mutation catalogue of mutation associated with drug resistance in *M*. *tuberculosis* [17], which covers a limited set of candidate genes (Table F in S1 Appendix) in the independent set. For first-line drugs, the catalogue had similar sensitivity (0.935 vs 0.936) with a higher specificity (0.992 vs 0.963) for isoniazid, and higher sensitivity (0.972 vs 0.960) and specificity (0.990 vs 0.984) for rifampicin. The machine learning system performed with much higher sensitivity for fluoroquinolones, with an increase in sensitivity for levofloxacin (91.2% vs 79.8%) and moxifoxacin (91.6% vs 85.9%), with slightly lower specificity (92.5% vs 95.1%, and 96.9 vs 95.1%, respectively). For amikacin, sensitivity was also higher (87.7% vs 85.1%), with lower specificity (94.9% vs 98.2%) (Table E in S1 Appendix).

As most patients in the world have little or no access to phenotypic DST, we also compared the performance of the machine learning system against the expected combined performance of Xpert MTB/RIF and Xpert XDR (molecular tests for rifampicin resistance, and for isoniazid, fluoroquinolone, ethionamide and aminoglycoside resistance, respectively) for the independent set, in anticipation of its wider uptake to address the WHO’s call for universal DST (Table G in S1 Appendix). The sensitivity of the machine learning system was 24% higher than Xpert for isoniazid (93.6% vs 91.1%, p<0.001) and rifampicin (96.0% vs 94.3%, p<0.001), 6% higher for moxifloxacin (91.6% vs 85.9%, p<0.001), and 2% higher for amikacin (87.7% vs 85.1%, p = 0.030). Specificity was no more than 1% lower for each drug, with the exception of isoniazid (96.3% vs 99.4%) and amikacin (94.9% vs 98.2%) (Table E in S1 Appendix).

## Discussion

We assess for the first time the extent to which machine learning can predict minimum inhibitory concentration of tuberculosis isolates based exclusively on whole-genome sequencing data for 13 antituberculosis drugs, including new and repurposed drugs. We use extreme gradient boosting, previously demonstrated to perform well on other bacteria and for binary tuberculosis prediction, and a set of unassembled genome-wide *k-*mers from sequencing reads instead of the more common approach of using genomic mutations. We followed best practice guidance for studies evaluating the accuracy of rapid tuberculosis drug susceptibility testing [18].

We find that our machine learning model can predict MICs with an essential agreement rate greater than 93% for first-line drugs, and 90% for other drugs. The WHO target product profiles for priority antituberculosis drugs, do not include thresholds and objectives for MIC prediction, focusing instead on binary metrics like sensitivity and specificity. However, essential agreements are similar to those reported for some other bacteria like *Salmonella* [10], *N*. *gonorrhea* [11], *S*. *aureus* [12], and *E*. *coli* [13] for which semi-quantitative data are more commonly generated.

MIC data could in the future help salvage the use of some drugs for some patients, by increasing in dose to overcome elevations in MIC that are only marginally above WHO-approved critical concentrations [19]. Much work need to be to identify how this might be done safely and effectively in the clinic, but being able to predict MICs from WGS data is a key step in that direction.

Current WHO guidelines for MDR-TB management recommend offering patients regimens including bedaquiline, linezolid, and potentially also clofazimine [19]. Our sensitivity and specificity for these three drugs fall below WHO TPP requirements. Our results reflect the very low prevalence of resistance to these drugs, which also explains the high negative predictive values and low positive predictive values. As more resistant isolates are collected, the sensitivity and specificity of the machine learning system will almost certainly increase, and negative predictive value decrease a little, as seen for other drugs. Nevertheless, a test with a high negative predictive value, lower positive predictive value, and sensitivity of 70% would still be helpful to clinicians [20]. Even imperfect test performance could still play a key role in preventing the amplification and dissemination of resistance.

Our system had higher sensitivity and negative predictive values on the independent test set compared to the expected performance of Xpert MTB/RIF and Xpert XDR for the drugs these probe, albeit at a small cost in specificity and positive predictive value. There are several explanations, including that the assays only look at eight genes and promoter regions, and exclude rare variants therein [5], while the machine learning system is able to explore genome-wide features, leverage interactions between features, and assess lineage and genetic background through genome-wide features. While we did not compare our approach to the new targeted next generation sequencing assays such as Deeplex MycTB (Genoscreen) [21], the ability to harness genome-wide information comprises a theoretical advantage over such assays.

WGS is likely to be more scalable than phenotypic drug susceptibility testing, with results for all drugs being derived from just a single test. Potential benefits of our approach to the analysis of WGS data include the ability to update and train automatically as new resistant samples are added. As resistance to drugs like bedaquiline emerges, this could help avoid the expensive multi-phase multi-year period required for the development of molecular assays [22,23], or the need to update catalogues through expert review [16]. The U.S. Food and Drug Administration recently released a regulatory framework for ‘live’ modifications to artificial intelligence and machine learning-based software as a medical device [24], and has recently provided clearance or approval for several such diagnostic devices [25], paving the way for clinical implementation and dissemination. Further advantages of our approach include that predictions are made for all isolates, avoiding the exclusion of 4–10% of samples with unknown mutations in candidate genes [17]. The use of *k*-mers from sequencing reads allows for genome-wide analysis whilst avoiding some of the vulnerabilities associated with read mapping or variant calling that can affect resistance prediction [26]. Whereas sites called with low confidence are filtered out of post-pipeline WGS outputs, our machine learning approach uses all *k*-mers from reads prior to processing by a pipeline as training features. Finally, by using an interpretable supervised machine learning algorithm, we provide a list of features used for prediction, which in turn can be hypothesis-generating in the search for causal mutations.

We note several limitations to our study. First, we were unable to validate essential agreement of MICs outside of the CRyPTIC dataset, due to the unavailability of such data (CRyPTIC is the first study to systematically collect MIC data across so many drugs). While we validate against binary phenotypes derived from MGIT, a priority of future work should be to perform external validation of MICs when such data becomes available. Second, there were a very small number of isolates phenotypically resistant to bedaquiline, linezolid, delamanid, and clofazimine in the CRyPTIC dataset. A third limitation is the use of a previously published literature-derived catalogue, rather than either of the more recently published WHO-endorsed catalogues [16], because the WHO catalogue of mutations associated with drug resistance in *M*. *tuberculosis* was developed using samples from both the CRyPTIC and independent datasets. It should be noted here that resolving any contradictions between catalogue-based predictions and ML predictions are intrinsically fraught, as ML predictions are not based on causality. Fourth, we report the performance of Xpert MTB/RIF and Xpert XDR *in silico*, but clinical performance of the actual tests might differ.

In summary, this study demonstrates that WGS can be combined with simple machine learning algorithms to provide MIC predictions for many drugs recommended for the treatment of susceptible and of MDR-TB. This study shows how a composite machine learning system could be used to help guide therapy, whilst being straightforward to updated as increasing numbers of resistant samples to new and repurposed drugs are collected. Further data and external validation are required before clinical implementation.

## Methods

### Ethics statement

Approval for CRyPTIC study was obtained by Taiwan Centers for Disease Control IRB No. 106209, University of KwaZulu Natal Biomedical Research Ethics Committee (UKZN BREC) (reference BE022/13), University of Liverpool Central University Research Ethics Committees (reference 2286), Institutional Research Ethics Committee (IREC) of The Foundation for Medical Research, Mumbai (Ref nos. FMR/IEC/TB/01a/2015 and FMR/IEC/TB/01b/2015), Institutional Review Board of P.D. Hinduja Hospital and Medical Research Centre, Mumbai (Ref no. 915-15-CR [MRC]), scientific committee of the Adolfo Lutz Institute (CTC-IAL 47-J / 2017) and the Ethics Committee (CAAE: 81452517.1.0000.0059) and Ethics Committee review, Universidad Peruana Cayetano Heredia (Lima, Peru) and LSHTM (London, UK).

### Study design

We performed a training, validation, and external testing study of a machine learning system to predict minimum inhibitory concentration (MIC) to 13 antituberculosis antibiotics using whole-genome sequencing data (WGS). We trained and tested the system on 10,859 isolates from 11 laboratories in 22 countries collected by the CRyPTIC consortium (Tables 1 and A in S1 Appendix). Phenotypes were determined using the UKMYC broth microdilution system [15]. We then assessed how this system, trained on UKMYC-derived phenotypes, would perform against a commonly used drug susceptibility testing method in independent samples. For this we made predictions for an external set of isolates used to derive the WHO catalogue of drug-resistant mutations [15]. We selected only those samples that had been phenotypically characterised by Mycobacteria Growth Indicator Tube (MGIT), namely 15,239 *M*. *tuberculosis complex* isolates from 22 countries (Table 1). All CRyPTIC and independent sample have been made public and are available for download [14] (https://ftp.ebi.ac.uk/pub/databases/cryptic/), and the list of isolates used for this study is presented in Tables H and I in S1 Appendix.

### Genotypic data

#### Whole-genome sequencing and k-mer generation

All isolates were whole-genome sequenced using Illumina next-generation sequencing, with sequencing protocols varying between sites as previously described [15]. Sequencing reads were trimmed and mapped to the reference genome H37Rv, and variants called using Clockwork (v0.8.3), a bespoke processing pipeline built for CRyPTIC and optimised to detect both single nucleotide polymorphisms and indels. Prior to mapping and calling, raw nucleotide *k*-mers from sequencing reads were set aside for training the machine learning predictor. In genomics, a *k*-mer refers to any string of *k* letters in a DNA sequence. The theoretical maximum number of possible k-mers in a DNA sequence of length L is 4L, since there are 4 possible letters at each position of the sequence (i.e. A, C, T, G, the four DNA nucleotides). When generating *k*-mers for a long DNA sequence, each possible overlapping k-mer is counted. Each read was decomposed into a series of 31-mers, in line with the standard for bacteria, and for feasibility reasons. While small values of *k* might generate *k*-mers that ambiguously map to many genome loci, large values of *k* will yield very specific *k*-mers that only occur in a few genes. Several studies found a *k*-mer length of 31 to be optimal for bacterial genome assembly [27,28]. 31-mers also offer considerable specificity and a manageable memory footprint [27,28], as they are the longest *k*-mer length that can be efficiently represented on a 64-bit machine. They have also been demonstrated to be superior to predict AMR with an AdaBoost model. Since *M*. *tuberculosis* is highly conserved, requiring fewer *k*-mer counts for a given specificity [28], *k*-mer counts of 31 are most appropriate for this analysis.

#### *K-mer* frequency correction and pattern generation

In order to use k-mers as features for machine learning algorithms, one must generate the list of k-mer features for each isolate. *k*-mers were generated in random order, and then reordered by alphabetical order of base pairs to facilitate analysis. While most *k*-mers are present more than 20 times in an isolate, some are present fewer than 5 times. This is likely the result of sequencing errors. One of the disadvantages of using *k*-mers from sequencing reads, as opposed to assembled genomes, is the lack of any error processing. For a given isolate, the frequency distribution of *k*-mer frequencies possesses two peaks: one at a frequency of 1, representing sequencing errors and *k*-mers present once, and another at a frequency of between 100 and 150, representing the true mode frequency. This is illustrated in S2 Fig where the *k*-mer distributions of fifty isolates are displayed, highlighting the variety of mode frequencies and standard deviations, and the presence of a peak at a frequency of 1 to 5 reads corresponding to sequencing errors (S2 Fig). To reduce the influence of these errors on our analysis, all *k*-mers present five times or fewer were removed from the dataset.

#### Pattern features and *k-mer* matrix generation

Given the number of unique *k*-mers in any dataset is several orders of magnitude greater than the number of samples, further processing is necessary to allow for efficient model training. We combined all groups of *k*-mers that always appear together across all isolates (that is, are present or absent in the same isolates) into a single feature. We refer to features that represent a combination of *k*-mers with the same presence or absence pattern as ‘patterns’ of *k*-mers. This procedure is a form of lossless compression, as no data is lost in the process of shrinking the feature space, and was originally described by Earle and colleagues [29]. We used a custom code developed by Earle and Wilson [29] to perform this pattern compression. First, all *k*-mer files are opened sequentially, and a single list of all unique *k*-mers present in the dataset is generated. Second, *k*-mers are combined in groups, and the pattern of presence or absence for all *k*-mers in the group is generated by interrogating each file successively. The *k*-mers present below a specified frequency threshold of 5 are discarded. Third, groups of k-mers generated in step 2 are combined, and all *k*-mers that follow the exact same pattern of presence or absence across the dataset are combined into a single pattern. The result is a list of patterns, and a key associating each pattern with a list of *k*-mers, allowing for feature analysis. Despite the effective and lossless compression provided by pattern combination, further file compression is required to generate *k*-mer matrices that can be effectively processed by machine learning algorithms. All *k*-mer feature matrices were stored and read using the Hierarchical Data Format. This is a file format system designed and developed to facilitate the management and storage of large data files, with less disk space and faster retrieve.

### Phenotypic data

#### Minimum inhibitory concentration phenotypes

Phenotypic drug susceptibility testing for the CRyPTIC training and test set was performed across all sites using a standard protocol described elsewhere [15]. Briefly, samples were subcultured and inoculated into 96-well broth microdilution plates containing 13 drugs; the plates were designed by the CRyPTIC consortium and manufactured by Thermo Fisher Inc., U.K. Between 5 and 10 doubling dilutions were used for each drug, and MICs for each were read after 14 days using three methods for quality assurance. MICs were converted to predictions of resistance or susceptibility using epidemiological cutoffs [15]. As the plate design was modified during the study, the intersect of both plates was used to determine the MIC, and concentrations outside both were right censored or left censored as appropriate. Phenotypic DST for the external test set used the BACTEC MGIT 960 system.

#### Phenotype processing

MIC values are both left and right censored. If the bacteria cannot grow in any of the wells, then the MIC must be lower or equal to that of the well with the lowest concentration and the value is left censored. If the bacteria can grow in all the wells, then the MIC must be greater than the highest well concentration and the value is right-censored. Several options can be explored to manage censored data when preparing the label vector. The first option would be to remove all censored phenotypes from the dataset. However, this would lead to a majority of samples being lost (>80% of isolates for rifabutin), especially since left censorship is expected in susceptible wild-type isolates when a drug is effective. The second option would be to replace all censored values with the value of the previous or next dilution. Finally, a maximum likelihood estimate could be used by fitting a multi-regression model to the data, preserving the uncertainty of censored MICs [30]. The challenge of censorship is compounded by the fact that the CRyPTIC dataset includes data from two different phenotypic plates, UKMYC5 and UKMYC6, each censored at different concentrations for each drug. To resolve the discordance between both plates, we only considered the intersection of both–concentrations that were present on both plates–and right or left censored the concentrations present on only one of the two plates (Table D in S1 Appendix). For simplicity and scalability, we then converted the censored values into the value of the previous or next dilution–where >1.6mg/L becomes 3.2mg/L. Finally, to account for the doubling dilutions and the sequential nature of MICs, we converted each value into its binary logarithm log_2_MIC. The resulting vector was used as the regression label vector.

### Model training

#### Hyperparameter tuning

For each model, we used a combination of best practices from the literature, grid search, and random search hyperparameter optimisation. In most cases we would use random search first to find the most impactful hyperparameters and initial values, and grid search for fine-tuning. We set aside an independent set of 1,000 isolates exclusively for hyperparameter tuning to avoid data leakage. We used 5-fold cross-validation with custom mean squared error scoring function. For random search, between 20 and 100 iterations were computed, depending on available computing power and time. In order to correct for class imbalance when evaluating models and performing feature analysis, we use stratified *k*-fold cross-validation for hyperparameter tuning. Samples are divided into *k* folds. One fold is used as the test set, while every other fold is used as the training set. Model performance and feature weights are computed, and the training starts again, using a different fold as the test set. This is repeated *k* times, until each fold has been used as a test set. After this process is complete, each sample has been used in the test set exactly once.

#### MIC prediction

Drug susceptibiluty for each sample was predicted using a *k*-mer-based, hypothesis-free, genome-wide supervised machine learning algorithm. Raw nucleotide *k*-mers (*k* = 31) from sequencing reads (i.e. prior to mapping or assembly) were used as features. A total of 1.9 x 109 individual *k*-mers were considered. Where <5 *k*-mers were identified for an isolate, these were considered sequencing errors (S2 Fig). We merged features across patterns [29], performed feature selection using the F-test applied to MICs, and trained an optimised tree-based extreme gradient boosting method to allow for rapid training, testing, and feature interpretation. After training, the top features relevant to each prediction were mapped to the H37Rv *M*. *tuberculosis* reference genome using bowtie2 for detailed feature analysis (S3 Fig and Table J in S1 Appendix). Youden’s J statistic was applied to derive the operating threshold of the system.

#### Computation

Given the large dataset size and high number of features, we explored ways to increase the speed of model training and computer memory to allow for training across the entire CRyPTIC dataset. Extreme gradient boosting methods generate an ensemble of learners sequentially, and not in parallel, with each new predictor attempting to correct the errors of its predecessors by minimising the residual function. As such, trees cannot be developed in parallel. However, the model’s system design enables it to compute in parallel through the use of a block structure. As the most consuming part of tree learning is getting the data into sorted order, Extreme Gradient Boosting stores the data in in-memory units called blocks. Different blocks may be distributed across cores and machines, or even out-of-core. A full description of the block design is presented in the first Extreme Gradient Boosting implementation paper [31]. All computations for machine learning training on *k*-mers were performed on remote cluster CPU nodes. We used the Biomedical Research Computer cluster of Oxford University, which includes over 7,000 CPU cores and 7 PB (one million GB) of fast, shared storage serving data at up to 30 GB/s. All computers run a Linux operating system. All Python jobs were sent to the cluster via ssh and a custom Univa Grid Engine scheduler. The cluster is composed of a series of computers (or nodes), with each node composed of cores. For processing the entire CRyPTIC dataset, we used a set of 3 Intel Ivybride Node with 48 cores per node and 41.25 GB of memory per core, for a total of 2 TB of memory available. We used the array job function when it was required to send hundreds of jobs across methods, drugs, and sample sizes in parallel.

#### Train-test

Performance on the 25% CRyPTIC test set was estimated by training the system on the 75% CRyPTIC samples not included in it. Performance on the independent test set was generated by training the system on the entire CRyPTIC dataset. P-values were calculated using McNemar chi-square test. We benchmarked the performance of the mutation catalogue and machine learning system against the expected performance of Xpert MDR/RIF and Xpert XDR (Cepheid, Sunnyvale, U.S.), based on the targets they probe (Table G in S1 Appendix). ‘Indeterminate’ predictions by the catalogue where a novel variation is seen in a candidate gene, were counted as susceptible for the purpose of the analysis. Finally, we simulated negative predictive values for each drug for different prevalence values of resistance. For each drug, we selected 138 samples at random to generate data sets with a percentage prevalence of resistance for every 1% between 1% and 49%, and repeated this 100 times. 138 corresponds to twice the number of isolates resistant to bedaquiline, the drug with the smallest resistance prevalence (69 resistant isolates).

## Author contributions

**Conceptualization:** David A Clifton, Timothy M Walker, Tim EA Peto, A Sarah Walker, Philip W Fowlder, Derrick W Crook, Alexander S Lachapelle

**Data curation:** Philip W Fowler, Zamin Iqbal, Martin Hunt, Daniel J Wilson, Sarah J Hoosdally, Ana Lúıza Gibertoni Cruz, Jeff Knaggs, Sarah Earle, Timothy M Walker, Alexander S Lachapelle

**Formal analysis:** Alexander S Lachapelle, Timothy M Walker

**Funding acquisition:** Camilla Rodrigues, David Moore, Derrick W. Crook, Daniela M. Cirillo, Zamin Iqbal, Nazir A. Ismail, Nerges Mistry, Stefan Niemann, Tim E.A. Peto, Guy Thwaites, A. Sarah Walker, Timothy M Walker, Daniel J. Wilson, David A Clifton

**Investigation:** The CRyPTIC Consortium

**Methodology:** Alexander Lachapelle, Timothy M Walker, Samaneh Kouchaki, Sarah G Earle, Yang Yang, David A Clifton

**Project administration:** The CRyPTIC Consortium

**Resources:** The CRyPTIC Consortium

**Software:** Alexander Lachapelle, Samaneh Kouchaki, David A Clifton

**Supervision:** Alexander S. Lachapelle, Timothy M Walker, David A Clifton

**Validation:** Alexander Lachapelle, Timothy M Walker

**Visualization:** Alexander Lachapelle

**Writing – original draft:** Alexander Lachapelle, Timothy M Walker

**Writing – review & editing:** The CRyPTIC Consortium

## Supporting information

S1 Fig Confidence matrices comparing MICs predicted by the machine learning system (X axis) with observed MICs (Y axis) for each drug. Drug names: INH: Isoniazid, RIF: Rifampin, EMB: Ethambutol, MXF: Moxifloxacin, LEV: Levofloxacin, AMI: Amikacin, KAN: Kanamycin, ETH: Ethionamid, RFB: Rifabutin. Other acronyms: MIC: Minimum inhibitory concentration.
(TIF)

S2 Fig The *k*-mer frequency distribution for a random subset of 50 MTB isolates from the CRyPTIC dataset, illustrating the error correction process for sequencing errors. S2 Fig Legend: The *k*-mer distributions of fifty isolates are displayed in blue, where the x-axis represents each *k-*mer count from 0 to 500, and the y-axis represents the frequency of that count in the isolate. For each each isolate there are two peaks–one peak at a frequency of 1, corresponding to sequencing errors, and one peak at a higher frequency (between 50 and 250), representing the actual peak. To reduce the influence of sequencing errors on our model training ad perforance, all *k*-mers present five times or fewer were removed from the dataset, as illustrated for four isolates at the top where *k*-mers in the red section of the graph were rejected, and *k*-mers in the green section were kept.
(TIF)

S3 Fig Illustration of the pipeline for *k*-mer feature analysis after machine learning system training. S3 Fig Legend: After the machine learning system was trained, a feature analysis was performed to identify potential mutations of interest. Since our machine learning features are patterns of *k*-mers, we follow four steps to translate patterns to mutations: (1) rank patterns by importance to the machine learning model based on their weight, (2) list all individual *k*-mers corresponding to each pattern, (3) map each *k*-mer to the reference MTB H37Rv genome to find its location, and (4) identify mutation for each pattern, and generate final feature list (see Table J in S1 Appendix).
(TIF)

S1 Appendix Supplementary Tables. (XLSX)

S1 File Membership of The CRyPTIC Consortium. (DOCX)
