# Supplementary figures and images for: Quantitative drug susceptibility testing for Mycobacterium tuberculosis using unassembled sequencing data and machine learning
Source: PLoS Comput Biol. 2024 Aug 5;20(8):e1012260. doi: 10.1371/journal.pcbi.1012260 (PMC11326700; doi:10.1371/journal.pcbi.1012260)

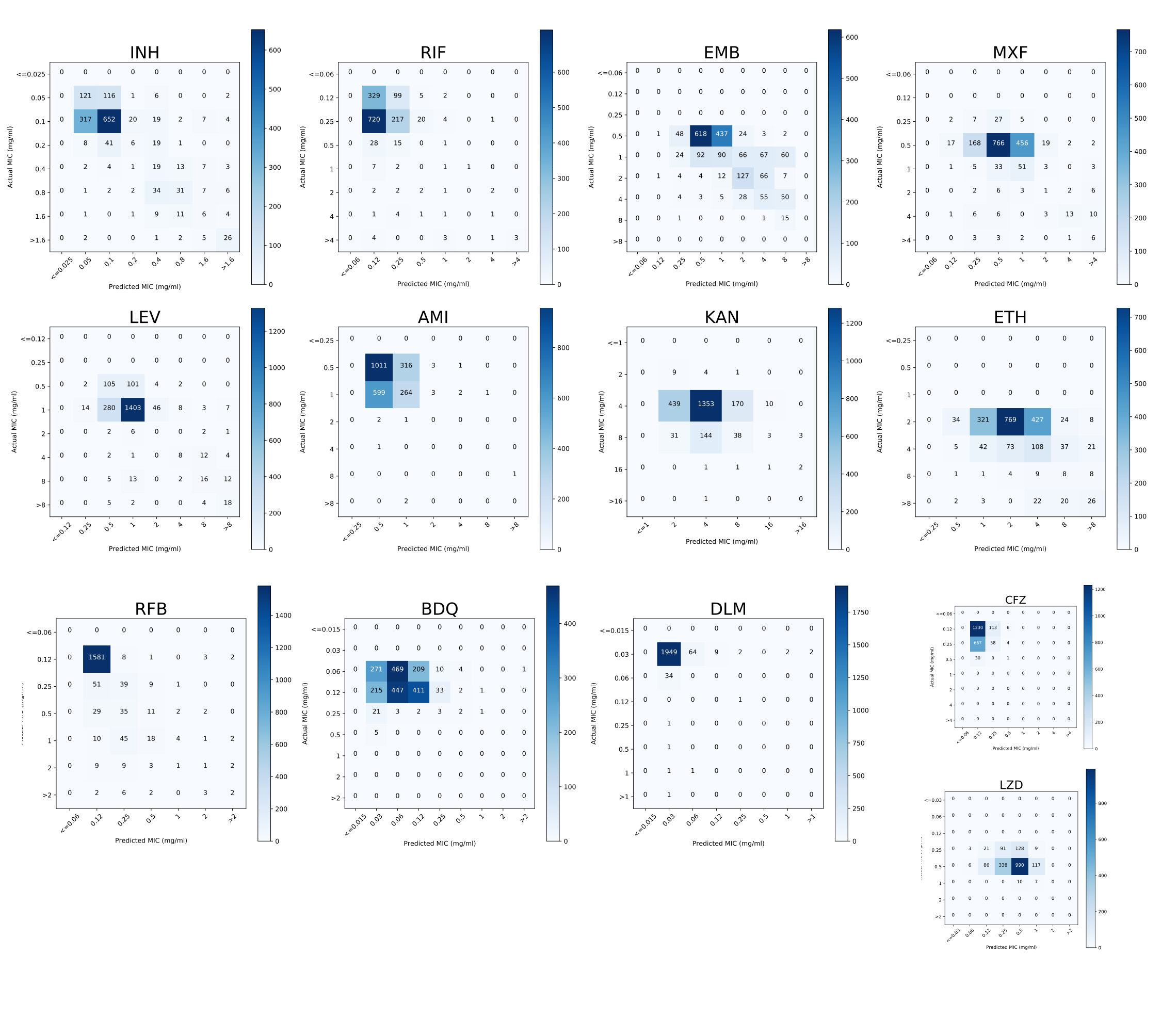

Supplement: S1 Fig — Drug names: INH: Isoniazid, RIF: Rifampin, EMB: Ethambutol, MXF: Moxifloxacin, LEV: Levofloxacin, AMI: Amikacin, KAN: Kanamycin, ETH: Ethionamid, RFB: Rifabutin. Other acronyms: MIC: Minimum inhibitory concentration. (TIF) [file pcbi.1012260.s001.tif]

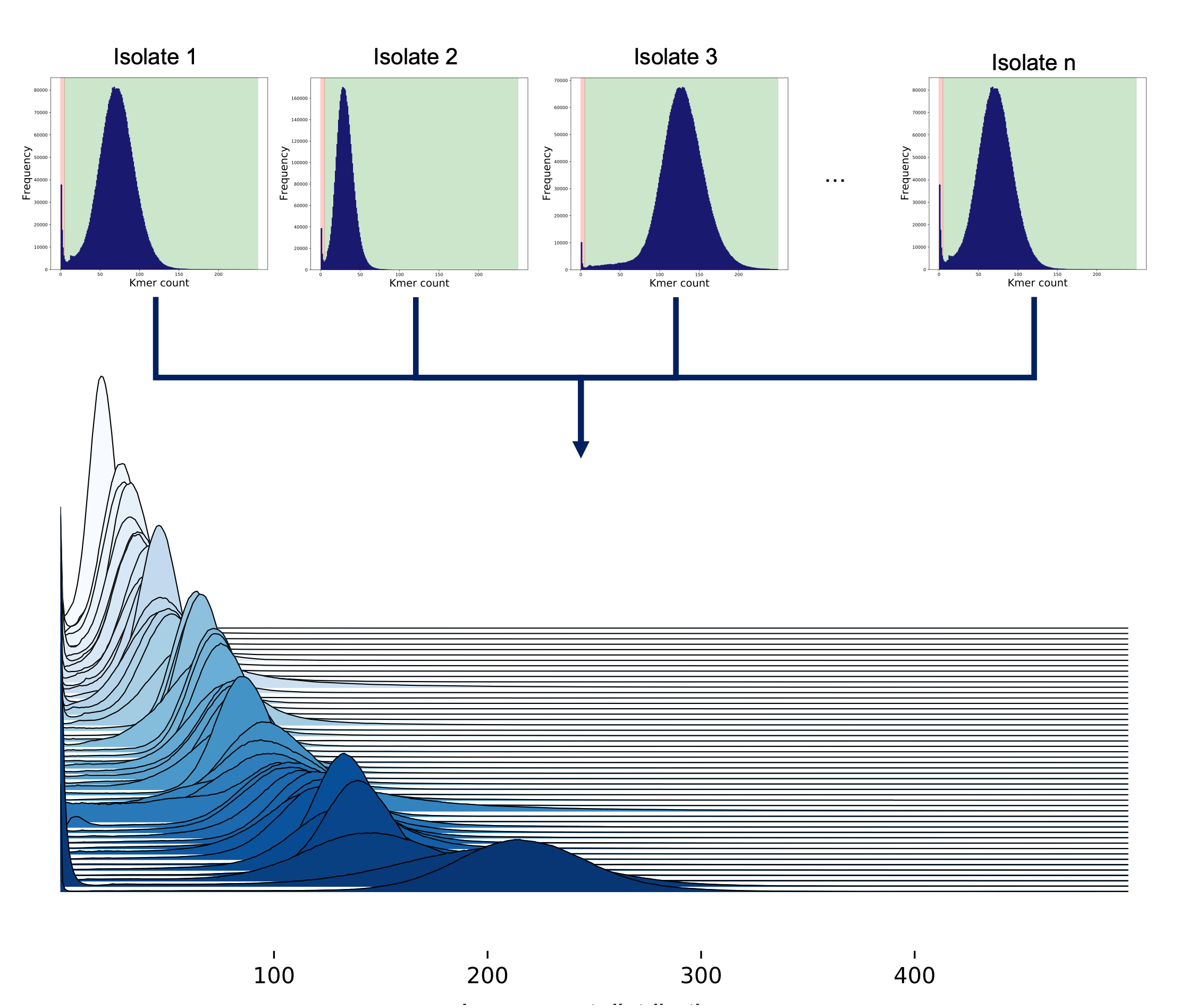

Supplement: S2 Fig — Legend: The k-mer distributions of fifty isolates are displayed in blue, where the x-axis represents each k-mer count from 0 to 500, and the y-axis represents the frequency of that count in the isolate. For each each isolate there are two peaks–one peak at a frequency of 1, corresponding to sequencing errors, and one peak at a higher frequency (between 50 and 250), representing the actual peak. To reduce the influence of sequencing errors on our model training ad perforance, all k-mers present five times or fewer were removed from the dataset, as illustrated for four isolates at the top where k-mers in the red section of the graph were rejected, and k-mers in the green section were kept. (TIF) [file pcbi.1012260.s002.tif]

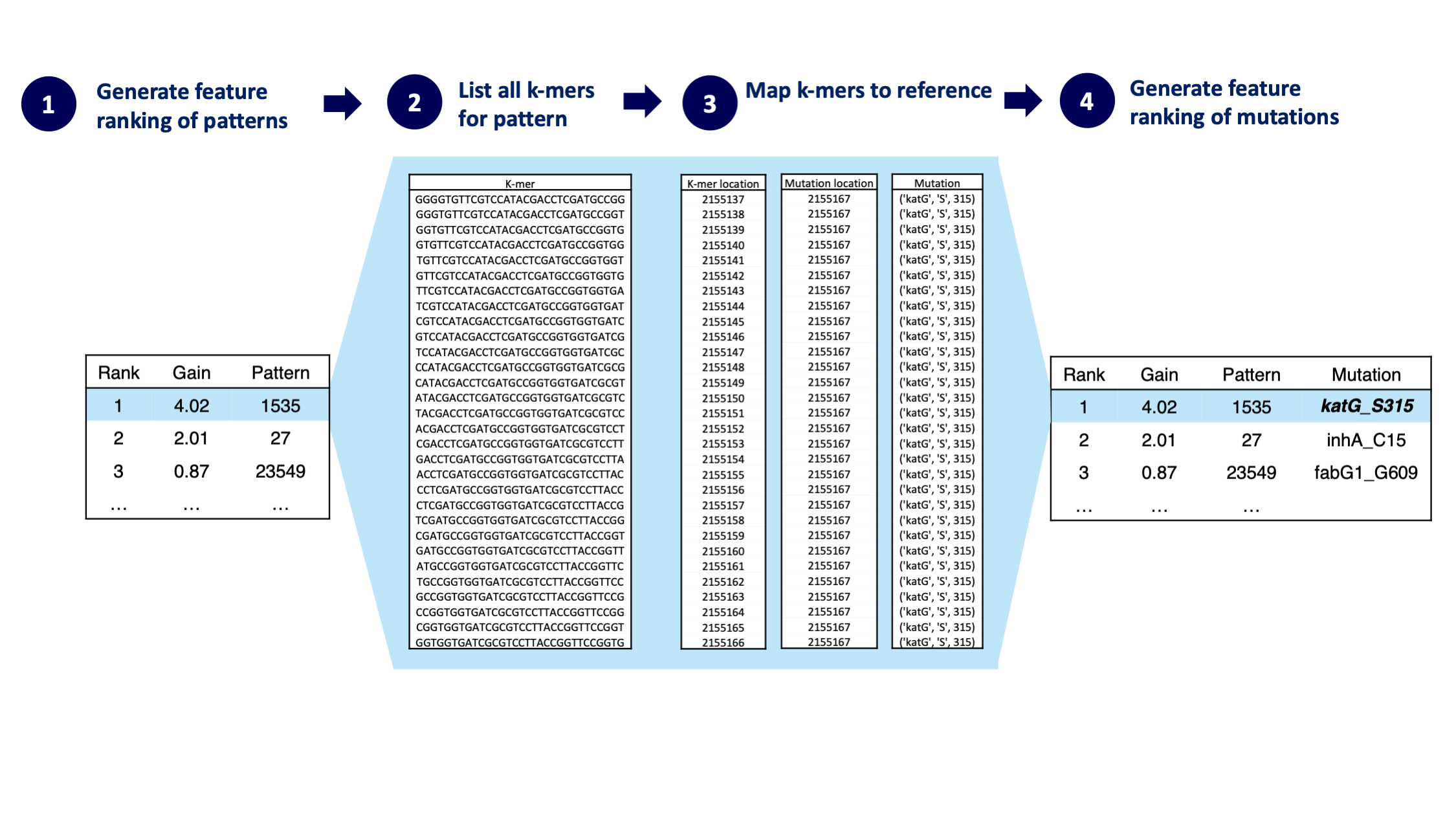

Supplement: S3 Fig — Legend: After the machine learning system was trained, a feature analysis was performed to identify potential mutations of interest. Since our machine learning features are patterns of k-mers, we follow four steps to translate patterns to mutations: (1) rank patterns by importance to the machine learning model based on their weight, (2) list all individual k-mers corresponding to each pattern, (3) map each k-mer to the reference MTB H37Rv genome to find its location, and (4) identify mutation for each pattern, and generate final feature list (see Table J in S1 Appendix). (TIF) [file pcbi.1012260.s003.tif]
