# Supplementary material for: Quantitative drug susceptibility testing for Mycobacterium tuberculosis using unassembled sequencing data and machine learning
Source: PLoS Comput Biol. 2024 Aug 5;20(8):e1012260. doi: 10.1371/journal.pcbi.1012260 (PMC11326700; doi:10.1371/journal.pcbi.1012260)
Supplement: S1 File — (DOCX) [file pcbi.1012260.s005.docx]

# Members of the CRyPTIC consortium (in alphabetical order)

Alexander S Lachapelle4,*, Ivan Barilar29, Simone Battaglia1, Emanuele Borroni1, Angela P Brandao2,3, Alice Brankin4, Andrea Maurizio Cabibbe1, Joshua Carter5, Daniela Maria Cirillo1, Pauline Claxton6, David A Clifton4, Ted Cohen7, Jorge Coronel8, Derrick W Crook4, Viola Dreyer29, Sarah G Earle4, Vincent Escuyer9, Lucilaine Ferrazoli3, Philip W Fowler4, George Fu Gao10, Jennifer Gardy11, Saheer Gharbia12, Kelen T Ghisi3, Arash Ghodousi1,13, Ana Luíza Gibertoni Cruz4, Louis Grandjean33, Clara Grazian14, Ramona Groenheit44, Jennifer L Guthrie15,16, Wencong He10, Harald Hoffmann17,18, Sarah J Hoosdally4, Martin Hunt19,4, Zamin Iqbal19, Nazir Ahmed Ismail20, Lisa Jarrett21, Lavania Joseph20, Ruwen Jou22, Priti Kambli23, Rukhsar Khot23, Jeff Knaggs19,4, Anastasia Koch24, Donna Kohlerschmidt9, Samaneh Kouchaki4,25, Ajit Lalvani26, Simon Grandjean Lapierre27, Ian F Laurenson6, Brice Letcher19, Wan-Hsuan Lin22, Chunfa Liu10, Dongxin Liu10, Kerri M Malone19, Ayan Mandal28, Mikael Mansjö44, Daniela Matias21, Graeme Meintjes24, Flávia F Mendes3, Matthias Merker29, Marina Mihalic18, James Millard30, Paolo Miotto1, Nerges Mistry28, David AJ Moore31,8, Kimberlee A Musser9, Dumisani Ngcamu20, Nhung N Hoang32, Stefan Niemann29, 48, Kayzad Soli Nilgiriwala28, Camus Nimmo33, Nana Okozi20, Rosangela S Oliveira3, Shaheed Vally Omar20, Nicholas I Paton34, Timothy EA Peto4, Juliana MW Pinhata3, Sara Plesnik18, Zully M Puyen35, Marie Sylvianne Rabodoarivelo36, Niaina Rakotosamimanana36, Paola MV Rancoita13, Priti Rathod21, Esther Robinson21, Gillian Rodger4, Camilla Rodrigues23, Timothy C Rodwell37,38, Aysha Roohi4, David Santos-Lazaro35, Sanchi Shah28, Thomas Andreas Kohl29, E Grace Smith21,12, Walter Solano8, Andrea Spitaleri1,13, Philip Supply39, Utkarsha Surve23, Sabira Tahseen40, Nguyen Thuy Thuong Thuong32, Guy Thwaites32,4, Katharina Todt18, Alberto Trovato1, Christian Utpatel29, Annelies Van Rie41, Srinivasan Vijay42, Timothy M Walker4,32, A Sarah Walker4, Robin M Warren43, Jim Werngren44, Maria Wijkander44, Robert J Wilkinson45,46,26, Daniel J Wilson4, Penelope Wintringer19, Yu-Xin Xiao22, Yang Yang4, Zhao Yanlin10, Shen-Yuan Yao20, Baoli Zhu47

**Institutions**

1 IRCCS San Raffaele Scientific Institute, Milan, Italy

2 Oswaldo Cruz Foundation, Rio de Janeiro, Brazil

3 Institute Adolfo Lutz, São Paulo, Brazil

4 University of Oxford, Oxford, United Kingdom

5 Stanford University School of Medicine, Stanford, California, United States of America

6 Scottish Mycobacteria Reference Laboratory, Edinburgh, United Kingdom

7 Yale School of Public Health, Yale, New Haven, Connecticut, United States of America

8 Universidad Peruana Cayetano Heredia, Lima, Perú

9 Wadsworth Center, New York State Department of Health, Albany, New York, United States of America

10 Chinese Center for Disease Control and Prevention, Beijing, China

11 Bill & Melinda Gates Foundation, Seattle, Washington, United States of America

12 UK Health Security Agency, London, United Kingdom

13 Vita-Salute San Raffaele University, Milan, Italy

14 University of Sydney, Sydney, Australia

15 The University of British Columbia, Vancouver, Canada

16 Public Health Ontario, Toronto, Canada

17 SYNLAB Gauting, Munich, Germany

18 Institute of Microbiology and Laboratory Medicine, IMLred, WHO-SRL Gauting, Germany

19 EMBL-EBI, Hinxton, United Kingdom

20 National Institute for Communicable Diseases, Johannesburg, South Africa

21 Public Health England, Birmingham, United Kingdom

22 Taiwan Centers for Disease Control, Taipei, Taiwan

23 Hinduja Hospital, Mumbai, India

24 University of Cape Town, Cape Town, South Africa

25 University of Surrey, Guildford, United Kingdom

26 Imperial College, London, United Kingdom

27 Université de Montréal, Montreal, Canada

28 The Foundation for Medical Research, Mumbai, India

29 Research Center Borstel, Borstel, Germany

30 Africa Health Research Institute, Durban, South Africa

31 London School of Hygiene and Tropical Medicine, London, United Kingdom

32 Oxford University Clinical Research Unit, Ho Chi Minh City, Viet Nam

33 University College London, London, United Kingdom

34 National University of Singapore, Singapore

35 Instituto Nacional de Salud, Lima, Perú

36 Institut Pasteur de Madagascar, Antananarivo, Madagascar

37 FIND, Geneva, Switzerland

38 University of California, San Diego, California, United States of America

39 Univ. Lille, CNRS, Inserm, CHU Lille, Institut Pasteur de Lille, U1019 - UMR 9017 - CIIL - Center for Infection and Immunity of Lille, Lille, France

40 National TB Reference Laboratory, National TB Control Program, Islamabad, Pakistan

41 University of Antwerp, Antwerp, Belgium

42 University of Edinburgh, Edinburgh, United Kingdom

43 SAMRC Centre for Tuberculosis Research, Stellenbosch University, Cape Town, South Africa

44 Public Health Agency of Sweden, Solna, Sweden

45 Wellcome Centre for Infectious Diseases Research in Africa, Cape Town, South Africa

46 Francis Crick Institute, London, United Kingdom

47 Institute of Microbiology, Chinese Academy of Sciences, Beijing, China

48 German Center for Infection Research (DZIF), Hamburg-Lübeck-Borstel-Riems, Germany

# Additional authors contributing to the CRyPTIC consortium (in alphabetical order)

Irena Arandjelovic1, Anna Barbova2, Maxine Caws3, Iñaki Comas4, Roland Diel5, Carla Duncan6, Sarah Dunstan7, Maha Farhat8, Margaret M Fitzgibbon9, Victoria Furió10, Jennifer Gardy11, Jennifer Guthrie6, Dang Thi Minh Ha12, Kathryn Holt13, Michael Inouye14, Frances B Jamieson6, SM Mostofa Kamal15, Julianne V Kus6, Vanessa Mathys16, Rick Twee-Hee Ong17, Youwen Qin7,14, Thomas R Rogers9,19, Gian Maria Rossolini20, Emma Roycroft9, Vitali Sintchenko21, Alena Skrahina22, Yik Ying Teo17, Phan Vuong Khac Thai12, Dick van Soolingen23, Mark Wilcox24, Matteo Zignol25

**Institutions**

1 University of Belgrade, Belgrade, Serbia

2 National Institute of phthisiology and pulmonology NAMS Ukraine, Kyiv, Ukraine

3 Liverpool School of Tropical Medicine, Liverpool, United Kingdom

4 Biomedicine Institute of Valencia IBV-CSIC, Valencia, Spain

5 University Medical Hospital Schleswig-Holstein, Kiel and Lübeck, Germany

6 Public Health Ontario, Toronto, Canada

7 University of Melbourne, Melbourne, Australia

8 Harvard Medical School, Boston,Massachusetts, United States of America

9 Irish Mycobacteria Reference Laboratory, Dublin, Ireland

10 Universitat de València, Valencia, Spain

11 Bill & Melinda Gates Foundation, Seattle, Washington, United States of America

12 Pham Ngoc Thach Hospital, Ho Chi Minh City, Vietnam

13 Monash University, Melbourne, Australia

14 Baker Institute, Melbourne, Australia

15 National Institute of Diseases of the Chest and Hospital, Dhaka, Bangladesh

16 Sciensano, Belgian reference laboratory for M. tuberculosis, Brussels, Belgium

17 National University of Singapore, Singapore

19 Trinity College Dublin, Dublin, Ireland

20 Careggi University Hospital, Florence, Italy

21 University of Sydney, Sydney, Australia

22 Republican Scientific and Practical Centre for Pulmonology and TB, Minsk, Belarus

23 National Institute for Public Health and the Environment, Bilthoven, The Netherlands

24 Leeds Teaching Hospital NHS Trust, Leeds, United Kingdom

25 World Health Organization, Geneva, Switzerland
